# SEXUAL FREQUENCY AND COGNITIVE DOMAINS: EVIDENCE FROM THE NATIONAL SOCIAL LIFE, HEALTH, AND AGING PROJECT

**DOI:** 10.1093/geroni/igad104.0899

**Published:** 2023-12-21

**Authors:** Shen Shannon

**Affiliations:** Hope College, Holland, Michigan, United States

## Abstract

This research draws upon the life course perspective to examine how sexual frequency is related to different cognitive domains among community-dwelling, partnered older adults. I analyzed data from 1,526 respondents across two rounds of the National Social Life, Health, and Aging Project (NSHAP). I tested six cognitive domains: temporal orientation, visuospatial skills, attention, language, executive function, and short-term memory. I ran cross-lagged models to test for reciprocal relationships between sexual frequency and cognitive domain performance. Age-stratified results indicate that more frequent sex was related to better performance on temporal orientation, executive function, attention, and short-term memory domains over time for adults aged 75-90, with no significant results for adults aged 62-74. There was limited evidence that cognitive domain performance was related to later sexual frequency for the 75-90 age group. The results highlight nuanced pathways by which sexuality is tied to the health of older adults. Understanding which cognitive domains may be maintained through more frequent sex is informative for health care practitioners who consider sexual activity as a behavior to prevent cognitive impairment.

